# Identification of significant features and machine learning technique in predicting helpful reviews

**DOI:** 10.7717/peerj-cs.1745

**Published:** 2024-01-23

**Authors:** Shah Jafor Sadeek Quaderi, Kasturi Dewi Varathan

**Affiliations:** Department of Information Systems, Faculty of Computer Science & Information Technology, Universiti Malaya, Kuala Lumpur, Malaysia

**Keywords:** Helpful reviews, Features, Review helpfulness, Machine learning, Online reviews, Random forest, SVM, Naive Bayes, Artificial neural network, Decision tree

## Abstract

Consumers nowadays rely heavily on online reviews in making their purchase decisions. However, they are often overwhelmed by the mass amount of product reviews that are being generated on online platforms. Therefore, it is deemed essential to determine the helpful reviews, as it will significantly reduce the number of reviews that each consumer has to ponder. A review is identified as a helpful review if it has significant information that helps the reader in making a purchase decision. Many reviews posted online are lacking a sufficient amount of information used in the decision-making process. Past research has neglected much useful information that can be utilized in predicting helpful reviews. This research identifies significant information which is represented as features categorized as linguistic, metadata, readability, subjectivity, and polarity that have contributed to predicting helpful online reviews. Five machine learning models were compared on two Amazon open datasets, each consisting of 9,882,619 and 65,222 user reviews. The significant features used in the Random Forest technique managed to outperform other techniques used by previous researchers with an accuracy of 89.36%.

## Introduction

The online platform has become popular among people as the most convenient product buying option in the last couple of years. After purchasing products online, many customers like to write their feedback about those products (O’Donovan et al., 2021; Alrababah, Gan & Tan, 2017). Therefore, thousands of reviews from users are continually being posted on e-commerce sites to share their opinions, and these reviews can be useful or otherwise (Saumya, Singh & Dwivedi, 2020; Xu, Li & Lu, 2019). A huge portion of consumers (around 97%) use online platforms to obtain detailed information about their desired products, and among them, 93% of consumers believe that online reviews influence their purchase decisions (Kaemingk, 2020). According to a survey from Brightlocal, nearly 59%–71% of United States internet users spent almost 15 min reading reviews before making their purchase decisions (Rimma, 2020). However, research shows that on average Americans spent USD125 per year making wrong purchase decisions based on these online reviews (Gaeta, 2020). The exponential growth of these online reviews generated by consumers has made the process tedious and difficult for readers in identifying helpful reviews. For instance, Yelp declared that its users provide 24,000 reviews per minute on their website (Shrestha, 2023). Therefore, it is essential to explore the attributes which detect helpful reviews (Almutairi, Abdullah & Alahmadi, 2019). If a review is considered helpful, it may provide more advantages in a customer’s product purchasing decision (Lee, Lee & Baek, 2021; Zhou & Yang, 2019), and assist in buying a quality product online (Eslami, Ghasemaghaei & Hassanein, 2018). A helpful review comprises additional product usage descriptions (Ren & Hong, 2019), personalized advice (Hu, 2020), description about pros and cons of that product (Chen et al., 2019), having good product rating (Malik, 2020; Topaloglu & Dass, 2021; Park, 2018), marked as helpful (Haque, Tozal & Islam, 2018; Qazi et al., 2016) by other users and contains adequate helpful votes (Anh, Nagai & Nguyen, 2019; Krishnamoorthy, 2015). Researchers in the past neglected lexical (Fan et al., 2018; Dewang & Singh, 2015), linguistic (Chen et al., 2019; Malik & Iqbal, 2018), and semantic features (Wang, Fong & Law, 2020; Kang & Zhou, 2019; Mukherjee, Popat & Weikum, 2017). Besides that, research has shown that neglecting the metadata features such as helpful votes leads to low performance on helpfulness prediction (Min & Park, 2012). Moreover, product rating is one of the metadata features that was not considered in helpfulness prediction which resulted in the degrading of the overall performance of review helpfulness prediction (Qazi et al., 2016). In addition, the preferences of those metadata features were not implemented properly in predicting the helpfulness of online reviews (Zhang et al., 2015).

The vast volumes of data (Malik & Hussain, 2018; Liu et al., 2017; Yang, Chen & Bao, 2016) raise the problem of information overload and are liable to predict low accuracy—72.82% (Anh, Nagai & Nguyen, 2019) in review helpfulness prediction model based on an open dataset. On the other hand, a citable performance in review helpfulness prediction has found 89.00% accuracy; however, a private dataset was used for that experiment (Momeni et al., 2013); thus, ambiguity has been created in review helpfulness models for dataset selection. Researchers extracted many features (Malik, 2020; Anh, Nagai & Nguyen, 2019; Malik & Hussain, 2018; Qazi et al., 2016; Krishnamoorthy, 2015) to predict the review helpfulness; nevertheless, in most cases, they selected the features by their preferences. The features selected using feature selection techniques are highly capable of precisely detecting whether a review is helpful or not helpful (Du et al., 2019). However, researchers hardly applied any features selections techniques to predict the review helpfulness based on the most suitable features, and as a result, the performances were not significant (Eslami, Ghasemaghaei & Hassanein, 2018; Zeng et al., 2014). Although many machine learning techniques have been applied to determine the performance of review helpfulness prediction; however, due to improper features utilization some researchers have obtained low performance (Anh, Nagai & Nguyen, 2019; Liu et al., 2017; Zhang et al., 2015; Zeng et al., 2014).

The objectives of this research are to identify the significant features using feature selection techniques from open datasets that contribute to determining the helpfulness of online reviews and use a suitable machine learning model by utilizing the identified features in predicting the helpfulness of online reviews. The rest of the article is organized as follows: ‘Related Works’ presents the related works of the study followed by ‘Methodology’ on research methodology. ‘Results & Discussion’ catered for results and discussions. Finally, ‘Conclusion & Future Work’ concludes the research with a summary of the findings, limitations, and future works.

## Related Works

The helpful reviews can assist consumers in the decision-making of online product purchasing, and 60% of consumers also believe that reviews are trustable regarding this purchasing decision (Bernazzani, 2020). A total of 76.5% of customers read less than 10 reviews during purchasing their desired products (Kavanagh, 2021). Since those customers skim a few reviews, there is a possibility of them overlooking the helpful reviews in making their purchase decision. This has led them to purchase a lower quality product online (Du et al., 2019). Customers tend to be influenced by the influence of some emotional or biased reviews in their purchase decision (Chen & Farn, 2020; Fresneda & Gefen, 2019; Malik & Hussain, 2018). Thus, helpful reviews greatly influence purchase decisions by ensuring the right choice in online shopping (Yang, Chen & Bao, 2016). This section illustrates the analysis of past research in predicting helpful reviews.

A significant number of researchers have used multiple types of machine learning techniques to determine the review’s helpfulness. The performance of review helpfulness prediction varies based on the techniques. Support vector machine (SVM) is a popular machine learning technique that was used by previous researchers in predicting the review’s helpfulness. Through the SVM, previous research determined notable performance (Krishnamoorthy, 2015; Martin & Pu, 2014; Zeng et al., 2014; Momeni et al., 2013; Min & Park, 2012; Weimer & Gurevych, 2007) where the highest accuracy was measured is 87.68% (Ghose & Ipeirotis, 2010). The Random Forest (RF) technique also showed significant outcomes (Son, Kim & Koh, 2021; Krishnamoorthy, 2015; Ghose & Ipeirotis, 2010), where the maximum accuracy was determined at 88.0% (Martin & Pu, 2014). Naïve Bayes is one of the standard techniques for review helpfulness prediction, and some researchers have made the review helpfulness prediction by Naïve Bayes (NB) with the highest accuracy of 85.0% (Krishnamoorthy, 2015; Martin & Pu, 2014). The decision tree (DT) technique measured maximum accuracy at 88.0% (Momeni et al., 2013) in review helpfulness prediction. Artificial neural network (ANN) is also used for review helpfulness prediction where the maximum outcome of 80.70% accuracy has been found (Eslami, Ghasemaghaei & Hassanein, 2018).

For predicting review helpfulness, regression techniques were also helpful. Using the tobit regression technique, the maximum Efron’s $R\hat {}2$ value-0.451 was measured (Korfiatis, García-Bariocanal & Sánchez-Alonso, 2012). Through the support vector machine regression technique, the highest Correlation Coefficient was measured at 0.712 (Zhang, Qi & Zhu, 2014) for helpfulness prediction. The researchers also used different types of techniques—Convolutional Neural Network (Olmedilla, Martínez-Torres & Toral, 2022), The recurrent neural network capsule (Anh, Nagai & Nguyen, 2019), multilayer perception neural network (O’Mahony & Smyth, 2010), rule-based classifier (Min & Park, 2012), linear regression (Zhang, Qi & Zhu, 2014), decision tree regression (Woo & Mishra, 2021), linear simple regression (Otterbacher, 2009), non-linear regression (Liu et al., 2008)—to predict the review helpfulness. However, this research has found the maximum performance among regression technique achieving accuracy of 89.0% (Momeni et al., 2013) was obtained by applying the Logistic Regression technique.

The performance of review helpfulness prediction is widely dependent on the available features in the dataset. This research has found that there are a good number of effective features’ categories that can enhance the performance of the technique for helpfulness prediction. The earlier research used different categories of features: linguistic features (Akbarabadi & Hosseini, 2020; Yang, Chen & Bao, 2016; Zhang et al., 2015; Susan & David, 2010), metadata features (Olmedilla, Martínez-Torres & Toral, 2022; Son, Kim & Koh, 2021; Qazi et al., 2016; Huang et al., 2015; Korfiatis, García-Bariocanal & Sánchez-Alonso, 2012), readability features (Menner et al., 2016; Momeni et al., 2013; Wu, Van der Heijden & Korfiatis, 2011), subjectivity feature (Ghose & Ipeirotis, 2010), polarity feature (Anh, Nagai & Nguyen, 2019; Liu et al., 2017; Zhang, Qi & Zhu, 2014), lexical features (Fan et al., 2018; Dewang & Singh, 2015), semantic features (Kang & Zhou, 2019; Mukherjee, Popat & Weikum, 2017; Liu & Park, 2015). Among those categories, lexical features are more sensitive (Novikova et al., 2019) and have simplification issues (Lee, 2010) in the extraction process; for that reason, those features/lexicons seem harder to extract easily (Qiu et al., 2009). Also, some low-dimensional features (*e.g.*, URL, @ Sign, Exclamation Marks, Hyperlinks) in the semantic feature category are less appropriate to represent the overall semantics in textual content (Cao, Wang & Gao, 2018). In addition, past researches show that the readability (Ghose & Ipeirotis, 2010) and subjectivity (Zhang & Varadarajan, 2006) features perform better than the lexical features to predict helpful review. However, linguistic features, metadata features, readability features, subjectivity features, and polarity features are easily extractable and received more popularity in review helpfulness prediction for wide usages in past research (Akbarabadi & Hosseini, 2020; Eslami, Ghasemaghaei & Hassanein, 2018; Krishnamoorthy, 2015; Martin & Pu, 2014; Wu, Van der Heijden & Korfiatis, 2011; O’Mahony & Smyth, 2010; Liu et al., 2008).

Product rating is one of the most used features for review helpfulness prediction. This metadata feature has been used widely by past researchers (Olmedilla, Martínez-Torres & Toral, 2022; Son, Kim & Koh, 2021; Woo & Mishra, 2021; Akbarabadi & Hosseini, 2020; Malik, 2020; Malik & Hussain, 2018; Menner et al., 2016; Qazi et al., 2016; Yang, Chen & Bao, 2016; Huang et al., 2015; Martin & Pu, 2014; Zeng et al., 2014; Min & Park, 2012; Korfiatis, García-Bariocanal & Sánchez-Alonso, 2012; Wu, Van der Heijden & Korfiatis, 2011; Ghose & Ipeirotis, 2010; Susan & David, 2010; Otterbacher, 2009; Weimer & Gurevych, 2007) in detecting helpful reviews. The review’s length is a valuable linguistic feature used by past research on review helpfulness prediction. Typically, a helpful review describes the details information of a product in a lengthy, and for that reason, a good review length (Akbarabadi & Hosseini, 2020; Malik, 2020; Eslami, Ghasemaghaei & Hassanein, 2018; Qazi et al., 2016; Yang, Chen & Bao, 2016; Yang et al., 2015; Zhang et al., 2015; Zeng et al., 2014; Wu, Van der Heijden & Korfiatis, 2011; Ghose & Ipeirotis, 2010; O’Mahony & Smyth, 2010; Susan & David, 2010; Liu et al., 2008; Weimer & Gurevych, 2007) can increase the performance of helpfulness prediction model, and for obtaining better outcome of the prediction model. Term Frequency is a notable linguistic feature of the review helpfulness prediction model. This feature extracts the word ratios from the textual content or review where the usage of the valuable words is counted and indicates the importance of a particular word within a review. On the other hand, term frequency is a primary component of the widely used TF-IDF technique. Many researchers (Olmedilla, Martínez-Torres & Toral, 2022; Liu et al., 2017; Menner et al., 2016; Yang, Chen & Bao, 2016; Yang et al., 2015; Zhang et al., 2015; Martin & Pu, 2014; Wu, Van der Heijden & Korfiatis, 2011) had used this Term Frequency for predicting review helpfulness and showed an effectual output. In addition, emotional word is also considered a useful linguistic feature by many researchers (Liu et al., 2017; Yang, Chen & Bao, 2016; Yang et al., 2015; Zhang et al., 2015; Min & Park, 2012; Wu, Van der Heijden & Korfiatis, 2011) in predicting the review helpfulness. On the other hand, eleven features are categorized as semantic features as shown in Table 1. The exclamation mark is one of the most popular emotional features that indicate surprise, anger, excitement, shock, delight, and fear. It also expresses precautionary statements such as danger, hazard, or unexpected event (Liu et al., 2017; Yang, Chen & Bao, 2016; Yang et al., 2015; Martin & Pu, 2014).

**Table 1:** Classification techniques used in predicting helpful reviews. Feature analysis in predicting helpful reviews.

Features	Author., year	Olmedilla, Martínez-Torres & Toral (2022)	Son, Kim & Koh (2021)	Woo & Mishra (2021)	Akbarabadi & Hosseini (2020)	Malik (2020)	Anh, Nagai & Nguyen (2019)	Eslami, Ghasemaghaei & Hassanein (2018)	Malik & Hussain (2018)	Liu et al. (2017)	Menner et al. (2016)	Qazi et al. (2016)	Yang, Chen & Bao (2016)	Huang et al. (2015)	Krishnamoorthy (2015)	Yang et al. (2015)	Zhang et al. (2015)	Martin & Pu (2014)	Zeng et al. (2014)	Zhang, Qi & Zhu (2014)	Momeni et al. (2013)	Korfiatis, García-Bariocanal & Sánchez-Alonso (2012)	Min & Park (2012)	Wu, Van der Heijden & Korfiatis (2011)	Ghose & Ipeirotis (2010)	O’Mahony & Smyth (2010)	Susan & David (2010)	Otterbacher (2009)	Liu et al. (2008)	Weimer & Gurevych (2007)
Linguistic features	Length of Sentence					X		X	X	X										X								X	X	
	Emotional Words									X						X	X	X					X	X						
	Term Frequency	X								X	X		X			X	X	X						X						
	Length of Review				X	X		X				X	X			X	X		X					X	X	X	X			X
	Uppercase Words																X									X				
	Verb’s Type																						X							
	Complemented Words																						X							
	Personal Information																								X					
	Number of Sentences in a Review												X					X		X				X		X		X		
	Describe Same Objects Repeatedly										X																			
	Describe about Name Entities																				X									
	Opinion Words																												X	
	Multiword Expression											X																		
	Number of Reviews of Review				X															X										
	Information of Products												X																	
	Information of Services												X																	
	Information of Price												X																	
	Number of Adjective and Adverbs								X											X										
	Complex Words																									X				
	Large Number of Syllabus																									X			’	
	Length of Character					X																								
Metadata Features	Rating	X	X	X	X	X			X		X	X	X	X				X	X			X	X	X	X		X	X	X	X
	Reviewer Identity			X	X								X	X											X					
	Marked as Helpful		X	X	X	X	X	X				X		X	X				X	X		X			X		X	X		
	Voted as Helpful		X	X	X	X	X					X		X	X				X	X		X			X		X	X		
	Reviewing Date		X	X	X	X						X			X					X		X						X	X	
	Title of Review	X		X	X	X			X													X								
Semantic features	Exclamation Marks									X			X			X		X												X
	Less Spelling Mistake																X								X					X
	Compare or “Adjective + er than”																		X											
	URL																													X
	Quote Function																													X
	Question Marks																													X
	Cue Words																						X							
	Less Non-English Words																								X					
	Higher Punctuation Marks																				X									
	More Hyperlink																				X									
	Sign @ for Replying																				X									
Lexical features	Topic Related Words										X								X		X									
	Pros and “Cons” String																		X											
	Effective Tense																						X							
	Describe Products’ Characteristics																								X					
	Describe Other Products as Alternate																								X					
	Topic Related Nouns, Verbs and Adjectives																	X						X						
	Topic Related Nouns and Verbs								X		X																			
Readability features	Readability				X				X						X			X			X			X	X	X		X	X	
Subjectivity feature	Subjectivity				X				X						X										X					
Polarity feature	Polarity of Sentence				X	X	X	X	X	X			X				X	X		X				X					X	

The lexical feature includes types of parts of speeches (especially noun, verb, and adjective words) in sentences (Yin et al., 2020; Hengeveld & Valstar, 2010). In user review, the Noun words represent the subject, and objects that indicate the products (Gordon, Hendrick & Johnson, 2004). The verb words refer to different actions (doing, feeling, working, *etc*.) that are product-oriented (Wilson & Garnsey, 2009), and the adjective words express the quality of those products (Jitpranee, 2017). Therefore, in earlier research, the topic related noun, verb, and adjective words were considered as citable features to determine review helpfulness (Malik & Hussain, 2018; Menner et al., 2016; Martin & Pu, 2014; Wu, Van der Heijden & Korfiatis, 2011). In addition, any words that are topic related and indicate the product’s characteristics and disadvantages are also used for review helpfulness measurement (Zeng et al., 2014; Momeni et al., 2013; Ghose & Ipeirotis, 2010).

The readability feature that represents the readability indices value indicates how easy and readable a review is. There are many readability indices such as the SMOG Index (Kasper et al., 2019), the Dale-Chall Index (DCI) (Singh et al., 2017), Coleman-Liau Index (CLI) (Liu & Park, 2015), Flesch Reading Ease (FRE) (Wu, Van der Heijden & Korfiatis, 2011), Gunning Fog Index (GFI) (Ghose & Ipeirotis, 2010), Flesch-Kincaid Grade Level (FKGL) (O’Mahony & Smyth, 2010), and Flesch-Kincaid (FK) (Agichtein et al., 2008). Many past researchers have considered this index as a valuable feature as shown in Table 1. On the other hand, the polarity feature shows the sentiment of a user review and this feature has proven to be useful in predicting helpful reviews. The least popular feature compared to lexical, linguistic, semantic, metadata, and polarity feature is subjective features. It is used to indicate the judgment of any person, shaped by his personal opinion and feelings instead of outside influences.

In terms of datasets used for predicting helpful reviews, Amazon datasets (Malik, 2020; Anh, Nagai & Nguyen, 2019; Malik & Hussain, 2018; Krishnamoorthy, 2015) are mostly preferred. In earlier review helpfulness prediction research, researchers used a large-sized dataset (Anh, Nagai & Nguyen, 2019), having 40 million reviews; however, due to insufficient features in the dataset, they obtained a low performance- 70.13% accuracy (Anh, Nagai & Nguyen, 2019). Choosing a suitable dataset that is open and has different types of features are useful for review helpfulness prediction (Malik, 2020; Malik & Hussain, 2018; Krishnamoorthy, 2015). Table 2 shows the open datasets which were used in predicting helpful reviews in past research. In addition, Table 3 depicts the performance evaluation of applied techniques in previous research.

## Methodology

**Figure 1:**
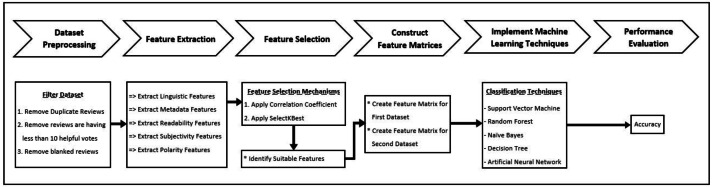
Methodology.

This research followed a customized framework for helpful review prediction by machine learning classification techniques from the open dataset. This framework comprises six stages; the initial stage is for data collecting from the dataset and preprocessing. Following the preprocessing, feature extraction and feature selection were performed. The next phase construct feature matrices which are used for training and testing data. Subsequently, the rest two stages for implementing machine learning techniques and, finally, the performances were evaluated. Figure 1 shows the research framework.

### Dataset

Two open available datasets got the preferences in this research. The first dataset was a multi-domain Amazon dataset (AD-MD) (Blitzer, Dredze & Pereira, 2007), consisting of 65,222 user reviews of DVDs, books, electronics, and kitchen & housewares. This dataset was also used in a few researches (Malik, 2020; Malik & Hussain, 2018; Krishnamoorthy, 2015). This dataset contains the highest number of features compared to other datasets used by past researchers. The second highest features contains in Kindle Review Amazon dataset (AD) from Kaggle with 9,885,619 user reviews on multiple products. Thus, this dataset is chosen as the second dataset for this research. These two datasets contain many similar features such as user reviews, product names, product types, reviewing date, title of the reviews, product ratings, and number of helpful votes.

**Table 2:** Feature analysis in predicting helpful reviews. Open dataset analysis in predicting helpful reviews.

**Dataset collected by/dataset name**	**Source**	**Available features**	**Number of reviews**
Blitzer, Dredze & Pereira (2007)	Amazon	Comments, Rating, Helpful Vote, Date, Title, Product Id, Product Name, Product Type, Reviewer Name, Reviewer Location	65,222
Jure Leskovec	Stanford University Data	Product Id, Product Title, Product Price User Id, Profile Name, Helpfulness, Score, Time, Summar, Text	34,686,770
Amazon Reviews for Sentiment Analysis	Kaggle	Comments, Rating	4,000,000
Kindle Review	Kaggle	Comments, Rating, Helpful Vote, Date, Title, Product Id, Product Name, Product Type	9,882,619

#### Normalize the target variable

For this research “Helpful Vote” was selected as the target variable. In this research, the threshold value was fixed at 0.60, which means, if a user review had more than 60% helpful votes, that review was considered a helpful review. This helpful vote’s threshold value was chosen based on the proven effectiveness in some previous research (Krishnamoorthy, 2015; Hong et al., 2012; Ghose & Ipeirotis, 2010). The helpful vote value is normalized to the binary value of 1 for review with more than or equals to 60% votes and 0 otherwise.

**Table 3:** Open dataset analysis in predicting helpful reviews. Performance of technique evaluation in predicting helpful reviews.

**Author., year**	**Dataset publicly availability**	**Technique**	**Performance matrices**	**Performance**
Olmedilla, Martínez-Torres & Toral (2022)	No	Convolutional Neural Network	Accuracy	66.00%
Son, Kim & Koh (2021)	No	Convolutional Neural Network	Accuracy	70.70%
Woo & Mishra (2021)	No	Tobit Regression	Accuracy	74.00%
Akbarabadi & Hosseini (2020)	No	Random Forest	Accuracy	85.60%
Malik (2020)	Yes^1^	Deep Neural Network	MSE	0.06
Anh, Nagai & Nguyen (2019)	Yes^2^	Convolutional Neural Network	Accuracy	70.13%
Eslami, Ghasemaghaei & Hassanein (2018)	No	Artificial Neural Network	Accuracy	80.70%
Malik & Hussain (2018)	Yes^3^	Stochastic Gradient Boosting	MSE	0.05
Liu et al. (2017)	No	Unigram Features + Argument-Based Features	Accuracy	71.80%
Menner et al. (2016)	No	Keyword Clustering	Accuracy	88.45%
Qazi et al. (2016)	No	Tobit Regression	Efron’s $R\hat {}2$	0.167
Yang, Chen & Bao (2016)	No	Support Vector Machine	Correlation Coefficient	0.665
Huang et al. (2015)	No	Tobit Regression	Efron’s $R\hat {}2$	0.128
Krishnamoorthy (2015)	Yes^4^	Random Forest	Accuracy	81.33%
Yang et al. (2015)	No	Support Vector Machine	Correlation Coefficient	0.702
Zhang et al. (2015)	No	Gain-based Fuzzy Rule-covering Classification	Accuracy	72.80%
Martin & Pu (2014)	No	Random Forest	Accuracy	88.00%
Zhang, Qi & Zhu (2014)	No	Linear Regression	Correlation Coefficient	0.712
Zeng et al. (2014)	No	Support Vector Machine	Accuracy	72.82%
Momeni et al. (2013)	No	Random Forest	Accuracy	89.00%
Korfiatis, García-Bariocanal & Sánchez-Alonso (2012)	No	Tobit Regression	Efron’s $R\hat {}2$	0.451
Min & Park (2012)	No	Rule-Based Classifier	Accuracy	83.33%
Wu, Van der Heijden & Korfiatis (2011)	No	Ordinary Least Square Regression	Correlation Coefficient	0.607
Ghose & Ipeirotis (2010)	No	Support Vector Machine	Accuracy	87.68%
O’Mahony & Smyth (2010)	No	Random Forest	AUC Score	0.77
Susan & David (2010)	No	Tobit Regression	Efron’s $R\hat {}2$	0.420
Otterbacher (2009)	No	Linear Simple Regression	Efron’s $R\hat {}2$	0.170
Liu et al. (2008)	No	Non-Linear Regression	F-Measure	71.16%
Weimer & Gurevych (2007)	No	Support Vector Machine	Accuracy	77.39%

**Notes.**

1,3,4 https://www.cs.jhu.edu/m˜dredze/datasets/sentiment1.

2 https://www.kaggle.com/datasets/bittlingmayer/amazonreviews.

3 https://snap.stanford.edu/data/web-Amazon.html.

### Tools and resources

The development of this review helpfulness prediction model has used Python as the programming language, and the preferred IDE was PyCharm. Furthermore, a couple of Python libraries were mandatory to complete the whole prediction model, such as like: numpy—for mathematical uses, pandas—for file manipulation, NLTK—for Natural Language Processing, textblob—for subjectivity and polarity score measurement, spacy—for genuine reviewer name and location validation, statistics—used for *Z*-score calculation, scipy—for correlation coefficient score calculation and sklearn—for machine learning techniques implementation and significant features selection. This research has used an Intel Core i7–7th generation computer with a CPU processing speed is 2.70 GHz–2.90 GHz. The other specifications of the used computer—240 SSD, 2TB HDD, 8 GB RAM. For the purpose of reproducibility, all source codes were documented and made available on GitHub: https://github.com/JaforQuaderi/Identification-Of-Significant-Features-And-Machine-Learning-Technique-In-Predicting-Helpfulness-Of-O.

### Preprocessing

Data preprocessing, one of the crucial steps in machine learning models (Ramírez-Gallego et al., 2017), is applied at the earliest stages of machine learning models to transform the data into a format that can easily be compatible with those models (Lawton, 2022). The suitable preprocessing methods may strongly affect the analysis of the performance of any machine learning model (Zhang et al., 2019). Data preprocessing methods help prevent overfitting (Nishiura et al., 2022), faster training and inference time (Jeppesen et al., 2019), eradicate data noise (Rahman, 2019), reduces redundant data (Zhang et al., 2021) and improve the accuracy (Kassani & Kassani, 2019; Makridakis, Spiliotis & Assimakopoulos, 2018) in machine learning models. Due to the significance of preprocessing, this research has applied the following filtering as data preprocessing:

a. Repeated reviews were removed—In those Amazon reviews datasets (AD-MD & AD), there are reviews those repeated twice or multiple times. To remove these duplicate reviews, a data deduplication method is applied. Initially, those datasets are converts convertedformat .csv format by using the build-in row repeating detection function; the repeated rows are identified and removed.
b. Consider reviews with at least 10 total votes—In those two datasets, many reviews have low total votes with higher helpfulness scores; those reviews seem less helpful for customers (Krishnamoorthy, 2015). For instance, a review that received six helpful out of eight total votes is considered less helpful than a review that received 22 helpful out of the 33 total votes. Therefore, this research has used reviews with at least 10 total votes to ensure the strength of the results, and strategy has also been applied in past research (Liu et al., 2008).
c. Empty reviews are removed—Empty reviews (*i.e.,* no textual content) with helpful and total votes are meaningless and ineffective. Hence, those empty or blank reviews are removed from those datasets.

### Feature selection

Researchers used a couple of techniques to determine the suitable features for the helpful review (O’Mahony & Smyth, 2010). The correlation coefficient is the most popular method to find out the significant features for review helpfulness (Kasper et al., 2019; Park, 2018; Yang, Chen & Bao, 2016; Yang et al., 2015; Zhang, Qi & Zhu, 2014; Wu, Van der Heijden & Korfiatis, 2011). In addition, arithmetic mean (Alrababah, Gan & Tan, 2017) and principal component analysis (PCA) & recursive elimination of features (RELIEF) (Zhang, Qi & Zhu, 2014) were used for this feature selection purpose. In addition, the SelectKBest is a built-in library in the Python programming language, which is another useful method for suitable feature selection (Anand et al., 2018).

### Feature extraction

For determining the review helpfulness from a certain dataset, it is obvious to extract the most relevant and significant features from that dataset. The feature selection can enhance any model’s performance prediction and helps to achieve better result (Gao et al., 2019). This research has preferred a significant number of features and identified the most useful features for this review helpfulness prediction model. Based on the literature review study, linguistic features, metadata features, readability features, polarity feature, and subjectivity feature were widely used feature categories for review helpfulness prediction, and therefore, this research selected these five feature categories.

#### Extract linguistic features

##### Apply POS tagging.

Ten types of linguistic features—noun, adverb, adjective, review length, number of sentences in a review, number of uppercase words, and four types of verbs—state verb, state action verb, interpretive action verb, and descriptive action verb—preferred in this research initially. The Linguistic Category Model (LCM) categorical operation was implemented through POS Tagging (Bird, 2006) to detect different linguistic features, especially the parts of speeches. Through the NLTK POS Tagging function, the parts of speeches are separated from each other, and this research divided parts of speeches into two major types—verb words and non-verb words. The preferred verb types—state verb, state action verb, interpretive action verb and, descriptive action verb. The preferred non-verb parts of speeches of this experiment are—noun, adverb, and adjective words.

##### Derive POS.

Through the NLP POS Tagging the verb, noun, adjective, and adverbs had identified. In the initial phase of Parts of Speech deriving, the different forms of verbs—state verb, state action verb, interpretive action verb, and descriptive action verb—were identified. Following these equations, the different types of verbs were identified-

(1) \begin{eqnarray*}& & \text{Action Verb Score}= \frac{1}{K} \sum _{K=1}^{3}\text{Subjectivity Score}(\text{word},\text{pos}=\text{` verbs'})\end{eqnarray*}

(2) \begin{eqnarray*}& & \text{Action Verb Type}= \left\{ \begin{array}{@{}l@{}} \displaystyle \text{SAV'}, \text{if Action Verb Score (word)}\geq \tau 1 \\ \displaystyle \text{` IAV'}, \text{if}\tau 2\leq \text{Action Verb Score (word)}< \tau 1 \\ \displaystyle \text{` DAV'}, \text{if Action Verb Score (word)}< \tau 1.  \end{array} \right. \end{eqnarray*}

The calculated score of action verbs was computed using the mean value of the subjectivity score of the Top K number of Synsets where K is starting from 1 Eq. (1). In this research, the maximum Synset value was fixed between 1 to 3. After the action verb Synset score was computed, it was categorised into SAV, IAV and DAV Eq. (2). The values of parameters *τ*1 and *τ*2 were set to 0.6 and 0.1, respectively, to determine the different type of action verb from the user review (Krishnamoorthy, 2015). In NLTK, the identification tag for noun: NN, NNP, NNS, NNPS, adverb: RB, RBR, RBS and adjective:—JJ, JJR, JJS. To derive the noun, adverb and adjective, all of these forms were used precisely.

##### Calculate *Z*-score.

After this linguistic category features extraction from the user reviews, a weight or score measurement technique had followed, and this research study gave the high preferences on *Z*-Score formula to calculate the weight for each of the linguistic features for all reviews. The linguistic feature set for a review: State Verb, State Action Verb, Interpretive Action Verb, Descriptive Action Verb, Nour, Adjective, Adverb. Considering below reviews to determine their linguistic feature sets:

Example 1—“The lamb-steak, so delicious. It is also very popular”.

The linguistic feature set for this review, LnFtr = (1, 0, 0, 0, 1, 1, 1).

Example 2—“Bugatti Chiron is a fastest super-car, and it is an expensive sports-car also. Rich persons like this sports-car”.

The linguistic feature set for this review, LnFtr = (2, 0, 0, 0, 2, 0, 1).

Example 3—“I always take milk in the morning. The Milk Nutrition has amazed me. It is a very good food as Protein. The fact is milk makes an antibody in the human-body. Hence, I am highly recommending to take milk every day”.

The linguistic feature set for this review, LnFtr = (1, 1, 1, 2, 5, 1, 2).

For computing the *Z*-Score, it was apparent to calculate the Mean and Standard Deviation for all linguistic features particularly. The basic formula of Mean (Eq. (3)), Standard Deviation (Eq. (4)), and *Z*-Score (Eq. (5)) are given below:

(3) \begin{eqnarray*}\text{Mean of linguistic feature}= \frac{\text{Sum of that specific linguistic feature}}{\text{Number of reviews}} \end{eqnarray*}

(4) \begin{eqnarray*}\text{Standard deviation of linguistic feature}\nonumber\\\displaystyle \quad =\sqrt{ \frac{\sum _{k=0}^{k=\text{number of reviews}}({\text{Each linguistic feature' s individual value}}_{k}-\text{Mean of that linguistic feature})^{2}}{\text{Number of reviews}-1} }\end{eqnarray*}

(5) \begin{eqnarray*}Z\text{-Score of linguistic feature' s individual value}\nonumber\\\displaystyle \quad = \frac{\text{Each linguistic feature' s individual score}-\text{Mean of that linguistic feature}}{\text{Standard deviation of that linguistic feature}} .\end{eqnarray*}

For calculating the mean and standard deviation, this review helpfulness prediction model preferred the built-in library of Python. However, the formula had to be implemented manually for the *Z*-Score calculation for the reviews. In the below, the compute values of the mean, standard deviation and *Z*-score for those three examples are given

Mean = (1.33, 0.33, 0.33, 0.66, 2.66, 0.66, 1.33)

Standard Deviation = (0.58, 0.58, 0.58, 1.15, 2.08, 0.58, 0.58)

*Z*-score for Example 1’s review = (−0.57, −0.57, −0.57, −0.57, −0.80, +0.59, −0.57)

*Z*-score for Example 2’s review = (+1.15, −0.57, −0.57, −0.57, −0.32, −1.14, −0.57)

*Z*-score for Example 3’s review = (−0.57, +1.16, −0.57, +1.17, +1.25, +0.59, +1.16)

##### Measure review length.

The helpfulness of user review is highly associated with review length. Though a short review could be helpful for the user, according to the human nature perspective, a good review should have a good size. Typically, longer reviews have detailed descriptions of products and contain information regarding where and how the products are used specifically. Thus, review length is considered a useful feature in this research.

##### Count capitalized words in review.

The number of capitalized words is also another useful linguistic feature. Capitalized words are considered as one kind of signal for the readers. The capitalized words indicate to customers some key points about the products. Sometimes, reviewer expresses their feelings, experiences, and satisfaction/dissatisfaction through the capitalized words in their reviews, and for this significance, this feature, number of capitalized words in a review, had preference in research.

##### Count sentences in review.

A review could be comprised of a single sentence or a combination of multiple sentences. Based on the user perspective, a review will get more preferences to other users if that review consists of informative content with multiple sentences. Typically, a lengthy review seems quite helpful for the other customers due to having details descriptions of products. Thus, these features also denoted a significant feature for this experiment.

#### Extract metadata features

##### Check reviewer name existence.

The reviewer name existence values were fixed as binary values—0 and 1, based on their reviewer existence. There are many review texts in the dataset where reviewer names had not been found; therefore, for that case, the reviewer name existence value has been set as 0. If the reviewer name has been found in the review, the reviewer name existences value has been set as 1. Also, the reviewer’s genuine name validation was computed for an additional purpose, and it has a binary value. The Python Spacy “en_core_web_lg” library’s “PERSON” function comprises all possible human names. This library assisted in this research to identify a valid human name. If the reviewer name has been identified as a valid human name, the reviewer name existence validation has set 1; otherwise, if the reviewer name is available, however, that is a pseudonym like—Superman, in that case, reviewer name existence validation has set 0.

##### Check reviewer location existence.

This research also considered the reviewer’s location existence as an important metadata feature. The reviewer location existence is also computed as binary values: 0 and 1. If the reviewer location information has been found in the review, the reviewer location existences value has been set as 1; otherwise, the value of the reviewer location existences has set as 0. Similarly, the reviewer location has been checked through the “GPE” function of Spacy “en_core_web_lg” library which is having all real geographical location information. If the reviewer location is valid, the review location existence validation value has set to 1; otherwise, the reviewer location existence validation value has set to 0.

##### Product rating.

The product rating is the most popular feature in the review helpfulness prediction model. This rating helps customers to understand how a product works in real life before they purchase it. The rating is varied from 1 to 5, where 1 is the lowest rating and 5 is the highest rating. This research collected the product ratings from every review from the datasets.

#### Extract readability features

There are different types of readability indices available in readability features. Measuring the readable scores from a textual content review or document is the primary task of those indices. This research has preferred 10 different readability indices: Flesch-Kincaid Grade Level, SMOG, Gunning Fog Index, Automated Readability Index, Coleman-Liau Index, Flesch-Kincaid, Flesch Reading Ease, and Dale-Chall Index for calculating the readability scores.

#### Extract subjectivity feature

Subjectivity is the only element in the subjectivity feature which generates a score from the textual content. This research has used a Python library—Textblob to calculate the subjectivity score. The range of subjectivity score is from 0 to 1, where 0 means that the text is not subject-oriented, and on the other hand, 1 means the text is fully subject-oriented.

#### Extract polarity feature

Polarity is also the only element of the polarity feature that defines the orientation of the expressed opinion, and this feature also generates a score from the textual content as well as the user review. Through the Python library Textblob, this research measured the polarity scores from each review. The polarity is a score range between −1 to +1. A score of −1 means the review is a thoroughly negative concept review. On the contrary, +1 means a positive review. In addition, if the polarity score value is 0, which means the review is neutral.

### Feature selection

The feature selection phase was initiated after computing the dataset’s features’ score based on linguistic, readability, metadata, subjectivity, and polarity features. From those five feature types, 27 features were extracted from the Amazon (65K reviews) dataset for the dataset initially. In the related works section, this research found that the Correlation Coefficient has a widely used technique to select significant features from the dataset. Therefore, this research used the correlation coefficient through Kendall’s Tau method to observe the relation between 27 features with the target variable as “Helpful”. In addition, another feature selection technique (SelectKBest), a Python-based feature selection mechanism used to determine the significant features. Table 4 highlights the Correlation Coefficient and SelectKBest scores based on all features.

## Results & Discussion

Based on the literature review study, this research has selected 5 machine learning classification techniques widely used in past research: SVM, RF, NB, DT, and ANN. This research experiment has followed the 80:20 ratio on training and testing with 10-Fold Cross-Validation.

Machine learning techniques were implemented on all the features from the AD-MD and the AD dataset. Despite executing the experiment, no output was retrieved since much higher hardware settings were required. For that reason, we applied feature selection techniques to obtain significant features in this review’s helpfulness prediction. These feature selection techniques made a difference between past research (Krishnamoorthy, 2015) and this research. Krishnamoorthy (2015) used 12 features without implementing the feature selection technique, whereas this research selected an equal number of features (top 12 features from Table 4) using feature selection techniques to implement the machine learning techniques. Table 5 shows the comparison of features used by past research with the features used in this study.

**Table 4:** Performance of techniques evaluation in predicting helpful reviews. Comparison of the correlation coefficient and SelectKBest scores based on the features.

**Features**	**Correlation coefficient scores with helpful**	**SelectKBest scores with helpful**	**Feature category**
**Rating**	**0.279878**	**1455**.**2**	**Metadata**
**Reviewer Location Existence**	**0.199417**	**405**.**4025**	**Metadata**
**Reviewer Name Existence**	**0.183377**	**316**.**0375**	**Metadata**
**Subjectivity**	**0.143916**	**276**.**8457**	**Subjectivity**
**Polarity**	**0.141497**	**311**.**0764**	**Polarity**
**Length**	**0.118247**	**112**.**8417**	**Linguistic**
**Adjective**	**0.117354**	**118**.**9454**	**Linguistic**
**IAV**	**0.11467**	**117**.**5278**	**Linguistic**
**Noun**	**0.108986**	**95**.**1823**	**Linguistic**
**Adverb**	**0.107725**	**96**.**45396**	**Linguistic**
**Sentences in a Review**	**0.107396**	**90**.**1899**	**Linguistic**
**Capitalized Words**	**0.105275**	**95**.**15608**	**Linguistic**
SAV	0.09265	69.18848	Linguistic
ARI	0.083694	57.38236	Readability
RIX	0.080336	30.78826	Readability
LIX	0.076998	32.57184	Readability
FK	0.076388	31.86075	Readability
DCI	0.066897	34.63997	Readability
FKGL	0.060843	6.485602	Readability
SV	0.057572	36.28841	Linguistic
GFI	0.056262	5.642237	Readability
SMOGI	0.04897	49.93567	Readability
DAV	0.048794	28.46312	Linguistic
CLI	0.038327	34.69642	Readability
Reviewer Location Existence Validation	0.030635	10.22768	Metadata
Reviewer Name Existence Validation	0.022298	7.191998	Metadata
FRE	−0.07262	36.80358	Readability

**Notes.**

The bold scores indicate the selected features in this research.

**Table 5:** Comparison of the correlation coefficient and SelectKBest scores based on the features. Comparison of features used.

Feature types	Features used (Krishnamoorthy, 2015)	Features used (this research)
Linguistic	State Verb, State Action Verb, Interpretive Action Verb, Descriptive Action Verb, Adjective	Interpretive Action Verb, ** Noun, Adverb, Adjective, Review Length, Capitalized Words, Sentences in a Review**
Metadata	Reviewing Date, Product Release Date	**Review Name Existence, Review Location Existence, Rating**
Readability	Flesch-Kincaid Grade Level, SMOG Index, Gunning Fog Index, Automated Readability Index, Coleman-Liau	–
Subjectivity	Subjectivity Score	Subjectivity Score
Polarity	–	**Polarity Score**

**Notes.**

The bold values reflect the differences between the features employed in this research compared to previous research (Krishnamoorthy, 2015).

According to Table 5, there are some citable differences in feature selection. This research has added some additional linguistic features such as noun, adverb, review length, and number of capitalized words in a review and sentences in a review, and eradicated state verb, state action verb, descriptive action verb, and Automatic Readability Index. In past research (Krishnamoorthy, 2015), the review date and product release date had been used; however, this research has preferred review name existence, reviewer location existence, and rating as metadata features. On the other hand, this research did not prefer any readability index as a readability feature, whereas the previous research (Krishnamoorthy, 2015) preferred five readability indices. Moreover, the polarity feature has been preferred as an important feature in this research where this feature had not been used in past research.

Krishnamoorthy (2015) used 12 different features and implemented three machine learning techniques on the AD-MD dataset. Similarly, the top 12 features had been selected in this research also for the result analysis and five machine learning techniques were applied to the Amazon AD-MD dataset. However, the AD dataset has no information about the reviewer’s name and reviewer location. For that reason, these two features were not included in the AD dataset; therefore, the preferred 5 machine learning classification techniques were applied to 10 features for the AD dataset in predicting review helpfulness. Table 6 illustrates the performance comparison of past research (Krishnamoorthy, 2015) and this research.

**Table 6:** Comparison of features used. Performance comparisons.

Techniques	Amazon 72K reviews dataset accuracy in past research (Krishnamoorthy, 2015)	Amazon (65K reviews) Accuracy on 12 features	Amazon (9.8 Mil Reviews) accuracy on 10 features
Support Vector Machine	77.81%	83.59%	88.98%
**Random Forest**	**81.33%**	**84.51%**	**89.36%**
Naïve Bayes	71.27%	81.68%	84.75%
Decision Tree	—	77.58%	83.89%
Artificial Neural Network	—	67.13%	72.61%

**Notes.**

The bold scores state the highest accuracy achieved.

According to Table 6, the RF technique has obtained the maximum accuracy—84.51% in this research for the AD-MD dataset in predicting helpful reviews. This is the highest accuracy detected in this research based on this AD-MD dataset, and this accuracy is also the highest accuracy ever for this dataset. From the comparison perspective, the SVM accuracies had obtained 83.59% and 77.81% accuracy, and the NB had received 81.68% and 71.27% accuracy in this research and earlier research, respectively, based on the AD-MD dataset. In addition, more significantly, the RF had gained 84.51% accuracy, whereas the earlier research had received 81.33% accuracy. Therefore, according to the comparison, this research showed better performances by these 3 classification techniques. The additional techniques—DT and ANN also showed notable results, especially the performance of DT that technique had performed 77.58% accuracy, which is higher than earlier research’s NB’s performance. Another implemented technique—ANN was added for additional research purposes and tried to observe the performance of the Neural Network technique on those datasets. This ANN technique received 67.13% accuracy, which was a moderate score.

For justifying this research, it was apparent to implement the exact mechanism and technique in another dataset. From the comparison perspective, all the techniques performed better in the AD dataset than the AD-MD dataset in this research experiment. For the AD dataset, in this research, the highest performance for the helpfulness prediction had gained 89.36% accuracy by the RF, and the second-highest performance obtained 88.98% accuracy through the SVM. The DT and NB performed quite satisfactorily, their accuracy was 84.75% and 83.89%, respectively, and the Neural Network technique—ANN achieved 72.61%, which was considered good enough, and did better performance than earlier performance evaluation experiments. The most important fact is that all of these performances were better than the previous research (Krishnamoorthy, 2015).

Moreover, the RF has achieved the highest accuracy for both datasets. The reasons behind this high performance was that RF randomly generated multiple trees in parallel from subsets of datasets. The features in datasets were also selected randomly during the splitting of nodes. Since RF has designed based on the decision trees; thus, the feature scaling did not matter for this technique. As a result, there were no over-fitting issues raised during program execution. In addition, the RF technique employed the Feature Bagging method, which decreased the correlation between internally generated decision trees and increased the mean accuracy of predictions, increasing the overall performance of review helpfulness prediction for both datasets.

## Conclusion & Future Work

This research has developed a model to predict helpful reviews from user-generated data. The significant features were identified, machine learning techniques were implemented and the performance of the review helpfulness prediction model was evaluated. For predicting the features that contribute to determining helpful reviews from user-generated data, this research examined these features—product rating, review length, capitalized words, sentences in a review, subjectivity, polarity, reviewer name existence, reviewer location existence, noun, adjective, adverb, interpretive action verb—are the most influential factors for review helpfulness prediction. For determining the most suitable machine learning techniques, this research implemented five machine learning classification techniques: SVM, RF, NB, DT, and ANN. Among those techniques, Random Forest performed the best on both the datasets used for performance evaluation with an accuracy of 84.51% on the AD-MD dataset and an accuracy of 89.36% on the AD dataset.

Several aspects were not addressed in this review helpfulness prediction model; hence, those become the limitations in this research. First of all, the dissimilarity in the available contents in datasets; that means, in the AD-MD dataset, there was information about the reviewer’s name and reviewer location; however, in the AD dataset, there is no information regarding the reviewer’s identity. There is also a lack of online datasets which contain the same features as the AD-MD or AD datasets. Secondly, to avoid training and dataset overloading problems, the semantic and lexical features were not included in this research. Due to the limitation of resources, this research could not execute the performance of the review helpfulness prediction based on all features. Future work should be emphasized on overcoming all these limitations.

## Additional Information and Declarations

### Data Availability

The following information was supplied regarding data availability: The Multi-Domain Sentiment Dataset is available at: https://www.cs.jhu.edu/ mdredze/datasets/sentiment/index2.html. The Amazon reviews: Kindle Store Category are available at Kaggle:
https://www.kaggle.com/datasets/bharadwaj6/kindle-reviews. The source code is available at GitHub and Zenodo: - https://github.com/JaforQuaderi/Identification-Of-Significant-Features-And-Machine-Learning-Technique-In-Predicting-Helpfulness-Of-O. - Quaderi SJS. (2023). Source Code_Identification Of Significant Features And Machine Learning Technique In Predicting Helpful Reviews. Zenodo. https://doi.org/10.5281/zenodo.10057199.
